# Evaluation of Noninvasive Tools for Predicting Esophageal Varices in Patients With Cirrhosis at Tygerberg Hospital, Cape Town

**DOI:** 10.1155/2024/9952610

**Published:** 2024-09-11

**Authors:** Lawrence Kwape, Shiraaz Gabriel, Ahmad Abdelsalem, Penelope Rose, Lefika Bathobakae, Dale Peterson, Desiree Moodley, Mohammed Parker, Saadiq Moolla, Arifa Parker, Keatlaretse Siamisang, Christoffel Van Rensburg, Ernst Fredericks

**Affiliations:** ^1^ Division of Gastroenterology and Hepatology Department of Medicine Faculty of Medicine and Health Sciences Stellenbosch University and Tygerberg Hospital, Cape Town, South Africa; ^2^ Department of Paediatrics and Child Health Stellenbosch University and Tygerberg Hospital, Cape Town, South Africa; ^3^ Internal Medicine St. Joseph's University Medical Center, Paterson, New Jersey, USA; ^4^ Division of Pulmonology Department of Medicine Faculty of Medicine and Health Sciences Stellenbosch University and Tygerberg Hospital, Cape Town, South Africa; ^5^ Unit for Infection Prevention and Control Department of Medicine Stellenbosch University and Tygerberg Hospital, Cape Town, South Africa; ^6^ Division of Infectious Diseases Department of Medicine Stellenbosch University and Tygerberg Hospital, Cape Town, South Africa; ^7^ Department of Family Medicine and Public Health University of Botswana, Gaborone, Botswana

**Keywords:** compensated cirrhosis, esophageal bleeding, esophageal varices, liver stiffness-spleen size-platelet ratio

## Abstract

**Background:** In patients with cirrhosis, esophageal variceal hemorrhage (EVH) is a devastating consequence of portal hypertension (PH). Upper endoscopy is considered the gold standard for the detection and diagnosis of esophageal varices (EVs), despite being invasive and costly. This study was aimed at identifying and evaluating the diagnostic accuracy of noninvasive tools in predicting EVs in patients with compensated cirrhosis.

**Methods:** This cross-sectional study included 50 patients with compensated cirrhosis at the Tygerberg Hospital Gastroenterology Clinic in Cape Town between November 2022 and May 2023. We collected clinical, anthropometric, and laboratory data from patients' physical and electronic charts. All patients underwent an abdominal ultrasound, vibration-controlled transient elastography (VCTE) to assess liver and splenic stiffness, and upper endoscopy. In this comparative study, we evaluated the diagnostic accuracy of different noninvasive tools in detecting EVs in patients with compensated cirrhosis.

**Results:** Of the 50 patients included in the study, 30 (60%) were female and 20 (40%) were male. The patients' age ranged from 18 to 83, with a mean age of 46.6 years. Cirrhosis was mainly due to alcohol use (*n* = 11, 22%), hepatitis B virus (HBV) infection (*n* = 11, 22%), and autoimmune hepatitis (*n* = 10, 20%). The patients included in the study were divided into two subgroups: with (*n* = 34, 68%) or without (*n* = 16, 32%) EVs. Statistically significant differences were detected between groups in platelet count (PC), liver stiffness measurement (LSM), spleen stiffness measurement (SSM), portal vein diameter (PVD), bipolar spleen diameter (SBD), aspartate aminotransferase-to-platelet ratio index (APRI), fibrosis-4 index (FIB-4), platelet/bipolar spleen diameter ratio (PSR), liver stiffness-spleen size-platelet ratio (LSPS), liver stiffness-spleen stiffness-platelet ratio score (LS^3^PS), and spleen stiffness-spleen size-platelet ratio score (SSPS) (*p* < 0.001). The highest diagnostic precision was observed with SSM (96%), SSPS (96%), LS^3^PS (94%), LSPS (94%), PSR (94%), and PC (92%). SBD (88%), LSM (86%), APRI (82%), and FIB-4 (82%) had the lowest diagnostic accuracy.

**Conclusion**: SSM and SSPS have the highest diagnostic accuracy for predicting the presence of EVs in patients with compensated cirrhosis. LSPS, LS^3^PS, and PSR come second at 94%. We recommend SSM and SSPS in institutions with transient elastography equipped with the software necessary to measure splenic stiffness. We introduce and propose LS^3^PS as a novel composite score for predicting the presence of EVs in patients with compensated cirrhosis. Large-sample-size studies are needed to validate these prediction scores and to allow direct comparison with Baveno VII. These prediction tools can help clinicians avoid unnecessary endoscopic procedures in patients with compensated cirrhosis, especially in developing countries with limited resources such as South Africa.

## 1. Introduction

Cirrhosis is histologically characterized by regenerative nodules surrounded by fibrous bands secondary to chronic inflammatory liver injury triggered by chronic liver disease, leading to portal hypertension (PH) and end-stage liver disease [1]. Liver cirrhosis evolves from being asymptomatic (compensated cirrhosis) to symptomatic (decompensated cirrhosis). Decompensation is marked by the development of obvious clinical signs, with the most frequent being ascites, variceal bleeding, encephalopathy, and jaundice [2]. A hepatic venous pressure gradient (HVPG) > 5 mmHg indicates the presence of PH [3]. Clinically significant portal hypertension (CSPH) is defined as an HVPG ≥ 10 mmHg [4]. Esophageal variceal bleeding (EVB) typically occurs when the HVPG is > 12 mmHg [5].

A progressive increase in PH leads to esophageal varices (EVs) due to portosystemic collateral circulation and eventually EVB [6]. The EV formation occurs at a rate of 8% per year [4]. Once EVs form, they progress from small to large at a rate of 5%–12% per year [7]. The risk of EVB is 25%–35%, accounting for 80%–90% of bleeding episodes in these patients [7]. EVB is the main cause of morbidity and mortality in patients with cirrhosis, despite improvements in the efficacy of endoscopic, pharmacological, surgical, and radiologic techniques [8]. The reported 1-year mortality for EVB ranges from 14.1% to 57% [6, 9, 10]. The overall mortality of isolated EVB is 20% at 5 years, increasing to 80% in addition to other decompensations [2]. The mortality risk is particularly high when EVB is associated with acute kidney injury and/or concurrent bacterial infections [11].

Endoscopy is the gold standard for diagnosing EVs and EVB in patients with decompensated cirrhosis. In patients with compensated cirrhosis, however, there are no clear indications for endoscopic evaluation of EVs [2]. Therefore, patients may be exposed to unnecessarily expensive and invasive procedures. This may result in an increase in the workload of the already busy endoscopy units and an additional financial burden on patients with chronic liver disease. Due to the unpleasant nature of the endoscopic procedure, patients may be reluctant to undergo endoscopy and become discouraged. The procedure also carries rare but serious complications, including perforation and bleeding. The annual incidence of complications varies among studies [12–14].

Simple noninvasive tools/markers have been developed to identify EVs in low-risk patients to avoid unnecessary endoscopy. These markers are used in routine laboratory investigations, making their daily application easier. These noninvasive markers of PH include platelet count (PC), platelet/bipolar spleen diameter ratio (PSR), aspartate aminotransferase-to-alanine aminotransferase (AST-to-ALT) ratio (AAR), fibrosis-4 index (FIB-4), and aspartate aminotransferase-to-platelet ratio index (APRI). The APRI has been reported to predict significant fibrosis in chronic HCV infections [15]. The FIB-4 was developed to predict liver fibrosis in patients with HIV/HCV coinfection [16]. Studies worldwide, which included a variety of study populations and different etiologies of liver cirrhosis, have reported the controversial and doubtful utility of these markers in predicting the presence of EVs [17–20].

Vibration-controlled transient elastography (VCTE) is a noninvasive tool for measuring liver stiffness (LS) (liver stiffness measurement (LSM)) and spleen stiffness (SS) (spleen stiffness measurement (SSM)), which correlates with CSPH [21]. A meta-analysis demonstrated that LSM alone is not sufficiently accurate to diagnose EVs. The liver stiffness-spleen size-platelet ratio score (LSPS), a combination of three simple examination methods (LS, spleen size, and PC), has been established to accurately predict EVs in patients with cirrhosis [22]. The Baveno VII criteria state that compensated cirrhosis patients with LSM ≤ 15 kPa and/or PC ≥ 150 × 10^9^ L and SSM ≤ 40 kPa have a low probability of having high-risk EVs; therefore, they can safely avoid endoscopy [23, 24].

The major challenges of these noninvasive tools are wide variations in optimal cutoffs and lack of external validity. The paucity of local data makes it even more challenging to adopt. In this study, we aimed to identify and evaluate the diagnostic accuracy of noninvasive tools in predicting EVs in patients with compensated cirrhosis, using the local data.

## 2. Methods

### 2.1. Study Design and Participants

We conducted a cross-sectional study among patients attending a daily gastroenterology outpatient clinic at Tygerberg Hospital in Cape Town, South Africa, between November 2022 and May 2023. Eligible patients were adults aged ≥ 18 years with compensated liver cirrhosis, regardless of the etiology. Compensated cirrhosis was diagnosed based on clinical examination, biochemical and imaging findings, or histology (only if available from previous liver biopsy results). The exclusion criteria were as follows: body mass index > 35, significant ascites, severe cardiopulmonary disease, renal failure, acute illness, pregnancy or breastfeeding, previous splenectomy or liver transplantation, previous transjugular intrahepatic portosystemic stent shunt procedure, and current or history of hepatocellular carcinoma. From a sample frame of a list of all patients attending the gastroenterology clinic during the study period, we used a systematic random sampling of three participants each day. On each day, we randomly selected the first participant from the numbered folded pieces of paper. Subsequently, every third patient in the clinic attendees was selected until the desired sample size for the day was reached.

### 2.2. Ethical Considerations and Informed Consent

Written informed consent was obtained from each patient before participation in the study. Ethical approval was obtained from the Stellenbosch University Health Research Ethics Committee (Ethics Reference No. S22/07/128).

### 2.3. Data Collection and Procedures

We used a researcher-administered questionnaire to obtain information on the patients' age, sex, etiology of cirrhosis, previous EVs, EVB and EV banding, comorbidities, and medications. For anthropometric measurements, we calculated BMI as weight in kilograms (kg) divided by height in meters squared. We evaluated the hemoglobin level (Hb), total leukocyte count (WCC), PC, mean corpuscular volume (MCV), serum albumin, serum bilirubin, aspartate aminotransferase (AST), alanine aminotransferase (ALT), PT, INR, sodium, urea, and creatinine levels in all participants. Endoscopy was performed on all recruited patients using an Olympus or Fujinon video gastroscope with an endoscopic record of the EV grade, if present. EV grading was defined as Grades I, II, and III [25]. VCTE was used to obtain the LSM, SSM, and CAP score in all participants using the FibroScan 630 Expert [26]. All recruited patients underwent abdominal ultrasound conducted by a radiologist. The bipolar spleen diameter (SBD) corresponded to the largest diameter measured from the inferior most tip of the spleen to the highest splenic point along the diaphragm [27]. The diameter of the portal vein was measured in its extrahepatic portion at the hepatic hilum, where visualization was optimal, just before bifurcation at the widest part of the blood vessel. The average of three measurements was taken to minimize the measurement error. PVD measurement was performed using a 3.5-MHz curvilinear probes of the EDAN U2 prime edition and SONOSCAPE SSI-8000 ultrasound machines [28].

### 2.4. Calculation of Noninvasive Markers/Tools

The following formulas were used to calculate the evaluated noninvasive markers:

1. AAR = AST/ALT ratio [29].
2. FIB‐4 index = (year of age × AST)/(PLT × the square root of ALT) [16].
3. APRI = (AST/upper limit of normal) × 100/PLT 10^9^/L [30].
4. PSR = PC/SBD ratio [31].
5. LSPSLSMvalueSBD/PC [32].
6. SSspleensizeplateletratioriskscoreSSPSSSMvalueSBD/PC [33].
7. LS^3^PS = LS‐SS‐platelet ratio score = LSM (kPa) × SSM (kPa)/PLT 10^9^/L.

### 2.5. Statistical Analysis

All calculations were performed using SPSS software (Version 29.0 for Windows; SPSS Inc., Chicago, Illinois, United States). Univariate analysis was performed to determine the association of various clinical, laboratory, LSM, SSM, and ultrasonographic variables with the endoscopic presence of EVs using Student's *t*-test for continuous variables and the chi-square tests for categorical variables. Student's *t*-test was used for normally distributed data and the Mann–Whitney *U* test was used for nonnormally distributed data. The differences were considered statistically significant with a *p* value of less than 0.05. All variables were used in prediction scores for the presence of varices using logistic regression analysis (multivariate analysis) to identify relevant predictors. Receiver operating characteristic (ROC) curve analysis was used to evaluate the performance of significant noninvasive markers and scores. The area under the curve (AUC) was estimated and reported with a 95% confidence interval.

## 3. Results

**Figure 1:**
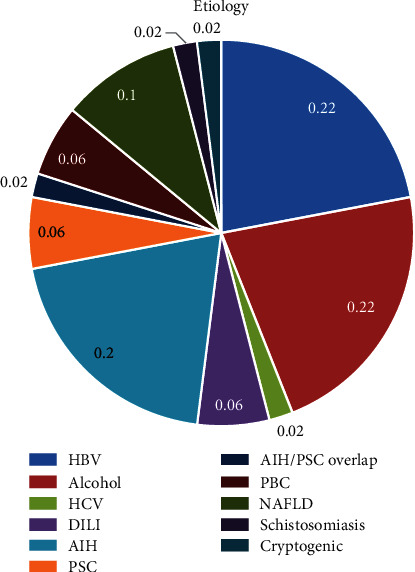
A pie chart demonstrates the various causes of cirrhosis in our studied population.

Fifty patients were recruited into the study from the 100 screened patients after excluding 50 ineligible patients. Demographic details are presented in Table 1. Thirty patients (60%) out of 50 were female, and 20 (40%) were male. The patients' age ranged from 18 to 83, with a mean age of 46.6 years. The most common causes of cirrhosis were found to be alcohol and hepatitis B viral infection, which accounted for 44% of cases (Figure 1).

**Figure 2:**
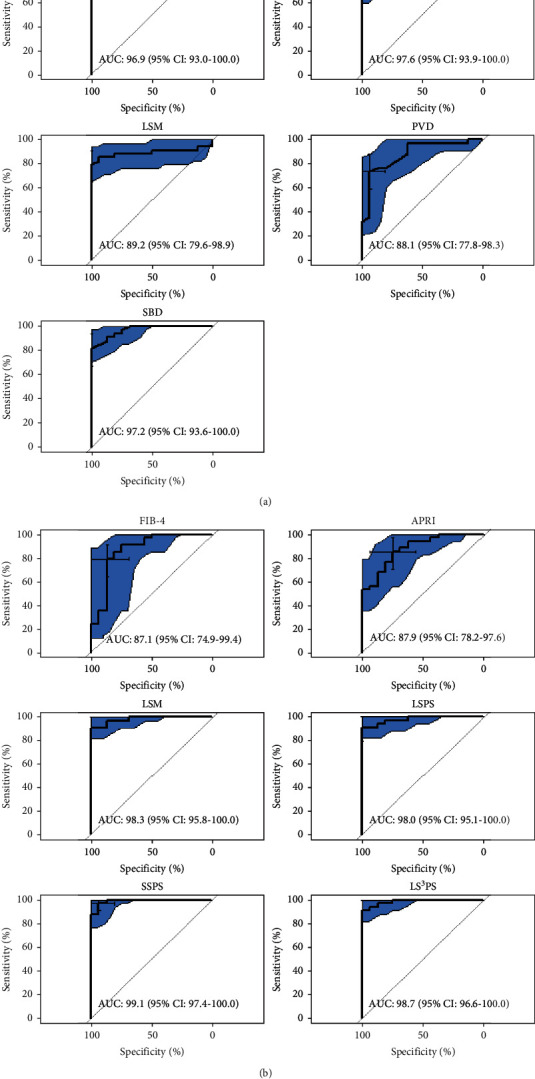
(a) ROC curve analysis was used to evaluate the performance of significant noninvasive markers. The AUC was estimated and reported with a 95% confidence interval. (b) ROC curve analysis was used to evaluate the performance of significant noninvasive scores. The AUC was estimated and reported with a 95% confidence interval.

Lower PC, all ultrasound and VCTE parameters, and all scores were associated with the presence of EVs in a statistically significant fashion (Table 2). SSM and SSPS scores had highest diagnostic accuracies followed by LS^3^PS, LSPS, and PSR as depicted in Table 3. Comparison between ROC curves of the various noninvasive tools is depicted in Figures 2(a) and 2(b).

## 4. Discussion

This outpatient cross-sectional study found PC, LSM, SSM, PVD, SBD, APRI, FIB-4, PSR, LSPS, LS^3^PS, and SSPS (*p* < 0.001) to be significantly associated with the presence of EVs. The highest diagnostic precision was observed with SSPS (96%) and SSM (96%). These were followed by LS^3^PS (94%), LSPS (94%), PSR (94%), PC (92%), SBD (88%), LS (86%), APRI (82%), FIB-4 (82%), and PVD (80%). PC was the most significant serum marker in the study. This study found that PC was significantly lower in cirrhotic patients with EVs than in patients without EVs. The PC cutoff value of < 145.1 had a sensitivity of 88%, a specificity of 100%, a PPV of 100%, an NPV of 80%, and a diagnostic precision of 92% in predicting the presence of EVs. Studies in Africa—Côte d'Ivoire, Egypt, and Uganda [34, 35]—showed similar results. This is also in agreement with multiple studies outside Africa, which mainly used a PC cutoff of < 100 with varying sensitivities, specificities, and diagnostic accuracies to predict the presence of EVs [31, 36–44]. The lower the PC, the higher the risk of EVs and EVB. Possible reasons for different cutoff values for PC could be varying degrees/severities of liver dysfunction as well as different etiologies for cirrhosis in different studies [45]. The etiology of thrombocytopenia in cirrhosis is multifactorial and includes platelet splenic sequestration due to PH and decreased thrombopoietin production, resulting in impaired platelet production. Thrombocytopenia may also be due to various medications used in the treatment of liver disease and its complications, including, but not limited to, immunosuppressants and antibiotics. Alcohol and certain viruses also have a bone marrow-suppressive effect [46].

Ultrasound findings associated with the presence of EVs included PVD and SBD (*p* < 0.001). Elastographic findings associated with EVs included LSM and SSM (*p* < 0.001). We found PVD to be significantly higher in patients with cirrhosis with EVs compared to those without EVs. A PVD cutoff value of ≥ 11.05 mm had a sensitivity of 74%, specificity of 94%, PPV of 96%, NPV of 63%, and diagnostic accuracy of 80% in predicting the presence of EVs. This is comparable to previous studies reporting PVD cutoff values from 11 to 14 mm with different sensitivities and specificities to predict the presence of EVs [47]. Variations in PVD could be explained by differences in study populations with varying etiologies and severity of liver cirrhosis, as well as different ethnic distributions [48].

Our study found that a higher SBD was significantly associated with the presence of EVs. SBD cutoff value of ≥ 121.9 mm had a sensitivity of 82%, specificity of 100%, PPV of 100%, NPV of 73%, and diagnostic precision of 88%. A study conducted in Nepal reported a sensitivity of 94.5% and a specificity of 75% for the prediction of EV presence using a 139-mm-diameter cutoff value for the spleen [49]. Increased PH leads to hypersplenism, resulting in splenomegaly [50]. The larger the diameter/volume of the spleen, the higher the risk of EVs.

This study found that patients with cirrhosis and EVs had a significantly higher LSM than patients with cirrhosis without EVs. An LSM cutoff value greater than or equal to 22.04 kPa had a sensitivity of 79%, specificity of 100%, PPV of 100%, NPV of 69%, and diagnostic precision of 86%. Our findings are comparable to those of a meta-analysis that included 44 studies carried out in Europe, Asia, Africa, and America. They found variable LSM cutoff values ranging from 6.1 to 29.7 kPa with high diagnostic accuracy (AUROC summary of 0.83) and a combined sensitivity of 82% and specificity of 68% for predicting the presence of EVs [51]. The literature reports a high variance in LS cutoff values, making it difficult to be used as a single tool to predict EVs; hence, the Baveno VII guidelines state that cirrhosis patients with an LSM ≤ 15 kPa and PC > 150 × 10^9^/L have a predictive value of more than 90% in excluding high-risk EVs that need intervention [24]. Different aims and approaches of the authors, as well as varying liver cirrhosis etiologies in various studies, could explain the variance in LS cutoff values [52]. LS is mainly the result of increased HVPG and high liver collagen content in patients with advanced cirrhosis [53].

The current study found that SSM values were significantly higher in cirrhosis patients with EVs than in those without EVs. An SSM cutoff value > 34.5 kPa had a sensitivity of 100%, a specificity of 88%, a PPV of 94%, an NPV of 100%, and the highest diagnostic precision of 96% in predicting the presence of EVs. This is in agreement with various studies reporting that SSM combined with other parameters, such as LSM, has high diagnostic accuracy for predicting the presence of EVs [54–56]. The current results are also consistent with work done in Germany that reported that SSM < 21.7 kPa was able to rule out CSPH with a sensitivity of 91.9% and SSM > 35.6 kPa was able to rule in CSPH, with a specificity of > 90% [57]. According to the Baveno VII criteria, SSM can be used in patients with advanced compensated liver disease, secondary to untreated HCV and HBV, to rule out and rule in CSPH (SSM < 21 kPa and SSM > 50 kPa, respectively) [24]. Splenic congestion due to PH causes architectural changes in the splenic arteries and veins, resulting in fibrosis and increased splenic stiffness [58].

This study evaluated various scores, including APRI, FIB-4, PSR, LSPS, LS^3^PS, and SSPS (*p* < 0.001), in predicting the presence of EVs in patients with compensated cirrhosis. The SSPS had the highest diagnostic accuracy in predicting the presence of EVs, followed by LS^3^PS, LSPS, and PSR. The APRI and FIB-4 scores demonstrated the lowest diagnostic accuracies. An SSPS cutoff value ≥ 2 had a sensitivity of 97%, a specificity of 94%, a PPV of 97%, an NPV of 94%, and a diagnostic precision of 96% in predicting the presence of EVs. There is a paucity of studies that evaluate SSPS in predicting EVs in patients with cirrhosis. Our work is comparable to a study in Korea that reported a sensitivity of 64.1%, specificity of 96.15%, and AUC of 0.85 using SSPS with a cutoff value of ≥ 3.70 to predict the presence of EVs in HBV cirrhosis patients [33]. SSPS is an additional emphasis that combining SSM, which is directly related to PH, with SBD and PC yields the highest diagnostic accuracy in predicting EVs, although more work is needed with larger sample sizes and broader cirrhosis etiologies.

**Figure 3:**
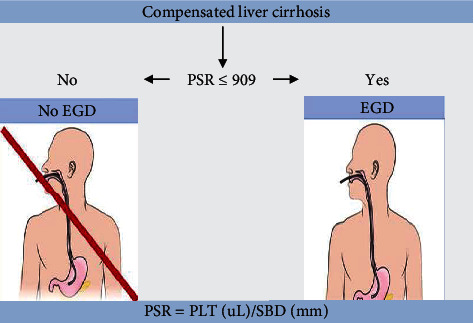
An illustration showing the application of PSR to predict EVs and need for endoscopy.

In our study, LSPS had a sensitivity of 91%, a specificity 100%, a PPV of 100%, an NPV of 84%, and a diagnostic precision of 94% using a cutoff value of ≥ 0.2 to predict the presence of EVs. This is comparable to the work done in Bangladesh, which reported that an LSPS cutoff value of ≥ 0.879 has 90.9% diagnostic accuracy in predicting EVs [22]. A meta-analysis of five studies across the world reported a pooled sensitivity of 69%, specificity of 86%, and AUC of 0.87 for LSPS in predicting the presence of EVs [59]. LSPS is a demonstration that combining LSM with SBD and PC further improves diagnostic accuracy instead of using LSM, SBD, or PC as a single tool for the prediction of EV presence. The current study found that the PSR score had a sensitivity of 91%, a specificity of 100%, a PPV of 100%, an NPV of 84%, and a diagnostic precision of 94% in predicting the presence of EVs at a cutoff value of < 1287.6. This is comparable to the findings reported in the meta-analysis of 49 articles done worldwide, including Africa, which presented a pooled sensitivity of 84%, specificity of 78%, and AUC of 0.87 using PSR cutoff < 909 (Figure 3) in predicting the presence of EVs [29].

**Figure 4:**
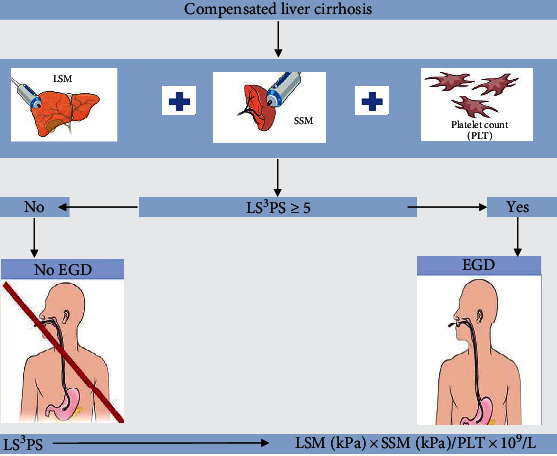
An illustration showing the application of new LS^3^PS to predict EVs and need for endoscopy.

LS^3^PS (Figure 4) had a cutoff value of > 4.96, a sensitivity of 91%, a specificity of 100%, a PPV of 100%, an NPV of 84%, and a diagnostic precision of 94% in predicting the presence of EVs. No studies have evaluated LS^3^PS for the prediction of EVs in patients with compensated cirrhosis. We formulated and tested the accuracy of LS^3^PS in our study for the first time and recommend it as a composite score for the prediction of EVs in compensated cirrhosis. LS^3^PS assessed the diagnostic accuracy of the combination of SSM, which is directly related to PH, with LSM and PC in predicting EVs. More studies with larger sample sizes and greater variation in cirrhosis etiologies are needed to validate the LS^3^PS score. This will also allow for a comparison with the currently used Baveno VII.

The APRI cutoff value of ≥ 0.91 had a sensitivity of 85%, a specificity of 75%, a PPV of 87%, an NPV of 71%, and a diagnostic accuracy of 82%, making it less reliable for predicting the presence of EVs. Our results are similar to those of previous studies conducted in other parts of the world. A study in Brazil demonstrated that an APRI cutoff value of > 1.3 has a sensitivity of 64.7%, specificity of 72.7%, PPV of 86.5%, and NPV of 43.2% to predict the presence of EVs, making it insufficient to independently predict the presence of EVs [60]. The APRI was initially described for the noninvasive prediction of fibrosis in patients with HCV [61]. In our study, the FIB-4 cutoff > 2.34 had a sensitivity of 79%, a specificity of 88%, a PPV of 93%, an NPV of 67%, and a diagnostic precision of 82% in predicting the presence of EVs. Our findings are consistent with a study in Egypt that reported that the FIB-4 cutoff value of > 2.78 has a sensitivity of 84% and a specificity of 86.67% in predicting the presence of EVs in patients with HCV cirrhosis [62]. The FIB-4 score was developed to predict liver fibrosis in patients with HIV/HCV coinfection [16].

To our knowledge, this is the first South African study to evaluate the precision of noninvasive tools in predicting the presence of EVs in patients with compensated liver cirrhosis. However, our findings should be interpreted with caution considering several limitations. The study cannot be generalized as it was a single-center study with a small sample size. An international study with a larger sample size is warranted to validate our prediction score. A larger sample size will also allow the comparison of all the prediction tools with the current Baveno VII.

## 5. Conclusion

In conclusion, this study demonstrated that SSM alone or in combination with spleen size and platelets count (SSPS) had the highest diagnostic precision in predicting the presence of EVs in patients with compensated cirrhosis. We therefore recommend SSM and SSPS in institutions with transient elastography equipped with the software necessary to measure SS (FibroScan 630 Expert). We introduce and propose LS^3^PS as a novel composite score for predicting the presence of EVs in patients with compensated cirrhosis. Large-sample-size studies are needed to validate these prediction scores and to allow direct comparison with Baveno VII. These prediction tools can help clinicians avoid unnecessary endoscopic procedures in patients with compensated cirrhosis, especially in developing countries with limited resources.

## Figures and Tables

**Table 1:** Demographic details.

**Variables**	**Total**	**(%)**	**With EVs**	**(%)**	**Without EVs**	**(%)**
	**50**		**34**	**68**	**16**	**32**
Gender: M:F	20:30	(40:60)	17:17	(50:50)	3:13	(18.7:81.3)
Age (years) [mean, SD]	46.6 ± 15.0		44.2 ± 14.5		51.8 ± 15.1	
BMI (kg/m^2^) [mean, SD]	25.1 ± 4.0		24.6 ± 3.5		26.1 ± 4.9	

Abbreviations: BMI, body mass index; EVs, esophageal varices; SD, standard deviation.

**Table 2:** Patient characteristics and univariate analysis.

**Variables**	**Total**	**Patients with EVs**	**Patients without EVs**	**Differences**
	**n** ** = 50**	**n** ** = 34**	**n** ** = 16**	**p** ** values**
Laboratory				
Platelet count (PC) (×10^9^/L) [median (IQR)]	123.0 (83.3–189.3)	92.0 (59.7–123.0)	218.0 (169.0–285.3)	< 0.001
Ultrasound				
Portal vein diameter (PVD) (mm) [mean, SD]	11.5 ± 2.6	12.6	9.1	< 0.001
Bipolar spleen diameter (SBD) (mm) [mean, SD]	125.3 ± 28.2	139.6	94.9	< 0.001
Transient elastography				
Liver stiffness (LSM) (kPa) [median (IQR)]	23.2 (18.8–38.8)	31.0 (22.8–48.4)	18.7 (16.1–20.0)	< 0.001
Spleen stiffness (SSM) (kPa) [mean, SD]	47.1 ± 21.3	57.3	25.3	< 0.001
Scores				
APRI [median (IQR)]	1.2 (0.6–2.3)	1.6 (1.1–2.8)	0.5 (0.3–0.9)	< 0.001
FIB-4 [median (IQR)]	3.0 (1.8–5.9)	4.5 (2.7–6.5)	1.4 (1.2–2.0)	< 0001
PSR [median (IQR)]	969.0 (540.6–1823.6)	615.0 (470.2–978.5)	2368.1 (1823.6–2543.9)	< 0.001
LSPS [median (IQR)]	0.4 (0.1–0.7)	0.6 (0.4–0.1)	0.1 (0.1–0.1)	< 0.001
LS^3^PS [median (IQR)]	14.6 (2.7–37.4)	25.9 (14.2–48.3)	1.9 (1.2–2.6)	< 0.001
SSPS [median (IQR)]	5 (1–9)	10 (5–10)	1 (1–1)	< 0.001

Abbreviations: APRI, AST-to-platelet ratio index; FIB-4, fibrosis-4 index; LSPS, liver stiffness-spleen size-platelet ratio; LS^3^PS, liver stiffness-spleen stiffness-platelet ratio score; PSR, platelet/bipolar spleen diameter ratio; SSPS, spleen stiffness-spleen size-platelet ratio score.

**Table 3:** Diagnostic performance of noninvasive tools for predicting esophageal varices.

	**AUC (95% CI)**	**Cutoff value**	**Sensitivity (%)**	**Specificity (%)**	**PPV (%)**	**NPV (%)**	**Diagnostic accuracy (%)**
Variables							
PC (×10^9^/L)	0.97 (0.93–1.0)	< 145.1	88	100	100	80	92
Ultrasound							
PVD (mm)	0.88 (0.78–0.98)	> 11.05	74	94	96	63	80
SBD (mm)	0.97 (0.94–1.0)	> 121.9	82	100	100	73	88
Transient elastography							
LSM (kPa)	0.89 (0.80–0.99)	> 22.04	79	100	100	69	86
SSM (kPa)	0.97 (0.94–1.0)	> 34.5	100	88	94	100	96
Scores							
APRI	0.88 (0.78–0.98)	> 0.91	85	75	87	71	82
FIB-4	0.87 (0.75–0.99)	> 2.34	79	88	93	67	82
PSR	0.98 (0.96–1.0)	< 1287.6	91	100	100	84	94
LSPS	0.98 (0.95–1.0)	> 0.2	91	100	100	84	94
LS^3^PS	0.98 (0.97–1.0)	> 4.96	91	100	100	84	94
SSPS	0.98 (0.95–1.0)	> 2	97	94	97	94	96

## Data Availability

Further inquiries can be directed to the corresponding author.
